# A Case of Amputation of the Thigh, in Which the Femoral Artery Was Successfully Compressed at the Groin

**Published:** 1803-06-01

**Authors:** G. Borrett

**Affiliations:** Surgeon, Great Yarmouth


### *A* Case *of* Amputation *of the* Thigh, *in which the Femoral Artery was successfully compressed at the Groin; by* G. Borrett, *Surgeon, Great Yarmouth.*

In Vol. V. No. xxii. of your useful Journal, I was favoured by your attention to a Paper on Strangulated Crural Hernia, which induces me now to beg the same indulgence, in your next Number, on the subject of Amputation.

February 7, Robert Grint, a strong healthy lad, fifteen years of age, while standing about a yard from the muzzle of a loaded gun, received its contents into his left thigh, at the place where the tourniquet is usually applied in amputation, which so dreadfully shattered the limb, that there appeared no prospect of saving it. I immediately called a consultation with my surgical friends, when amputation was considered the most judicious step to be taken; but as this operation involved difficulties new to us, us, much apprehension was entertained for its success, From the situation of the fracture and extent of the injury, no tourniquet could be applied with effect, and, but for the opinion delivered by Mr. Astley Cooper, when I attended his lectures in the year 1795, "*that any bleeding in the thigh and leg was under our command by pressure upon the femoral artery at the groin,*" the operation in this case might have been relinquished, or if performed, unsuccessful; for every man is not prepared to dash down upon a large artery and meet a torrent of blood, which, if not stemmed in a few seconds, destroys the patient.

At eight o'clock in the evening, (four hours after the accident,) every thing was ready for the operation, when the compression of the artery at the groin was assigned to Mr. Downes. The amputation was performed in the usual manner and much to our satisfaction, seeing that the compresion of the artery was judicious and effectual. The artery was tied by means of the tenaculum with a small ligature as usual after amputation. The limb was removed high up, leaving not more than three inches of bone below the trochanter major.

On the second evening after the operation, not a speck of blood was seen through the dressings; but early on the following morning I was called to my patient, in consequence of a sudden shew of blood the breadth of the whole face of the stump. The person who watched, instantly compressed the artery over the ramus pubis, in the manner he had been instructed, in case of such an appearance. Apprehending that there was no security for my patient any longer than the pressure could be steadily continued, I sent for my surgical friends, determined to lay bare the artery, and tie it an inch below the os pubis, which was easily accomplished. On the eleventh day from the application of this second ligature it came away; no hæmorrhage ensued in consequence of its separation, and all appeared well except a sphacelus, an inch in breadth, of the skin and cellular substance, on the edge of the fore part of the stump, which I imagined had come on in consequence of the artery being tied at the groin, and by that means the direct supply of blood to the skin on the fore part of the stump cut off.

After the limb was removed, it was examined; five splinters of bone (from one inch and a half to two inches long, and half an inch thick) were found; the femoral artery being lacerated and the muscles much torn.

In

In the second operation we had the most satisfactory demonstration that the artery may be effectually compressed at the groin; for, in exposing the vessel, I unluckily divided a cuticular branch, about the eighth of an inch from the main trunk, and the pressure at the groin being removed, the blood jutted out per saltum, but was immediately stopped by the pressure at the groin.

In No. xli. Vol. viii. of your Journal, Mr. A. Cooper, gives a paper on Aneurism, and in a short note observes, "Mr. Lucas amputated a thigh so near the groin no tourniquet could be applied, and the patient was secured from bleeding during the operation by pressure upon the artery at the groin." I have been induced to offer this case for publication to corroborate the above practical fact. If pressure at the groin may be relied upon in amputation, may it not also in the operation for femoral aneurism? Mr. John Bell, in his book on Surgery, vol. i. p. 414, under the head "of the difficulty of compressing the great arteries at the axilla or groin," observes, "in performing our operations on the great arteries of the arm-pit or groin. we must trust nothing to assistants, nothing to compression of the artery; we must do our operation with peculiar boldness and skill, otherwise we shall hardly save our patient, for in a single moment he is either safe or dead, &c."

This conclusion Mr. Bell has supported by some striking and well authenticated cases, which I do not mean to doubt or deny. They carry conviction to every mind as to the existing circumstances, but do not prove incontestibly, that, compression of the artery at the groin is unsafe; or that it is injudicious to avail ourselves of the practical fact which these two cases substantiate. I trust that they will have their use, by strongly impressing the minds of surgical gentleman on this important point in their profession.

I take this opportunity of expressing my sense of Mr. Edward Kentish's invaluable Essay on Burns; since the public was favoured with his Treatise, I have had several cases of scalds and burns, the easy and rapid healing of which have exceeded my expectations.

*May* 9, 1803.

COPY.

